# Exosomes Derived from Hypertrophic Scar Fibroblasts Suppress Melanogenesis in Normal Human Epidermal Melanocytes

**DOI:** 10.3390/ijms25137236

**Published:** 2024-06-30

**Authors:** Hui Song Cui, So Young Joo, Yoon Soo Cho, You Ra Lee, Yu Mi Ro, In Suk Kwak, Gi Yeun Hur, Cheong Hoon Seo

**Affiliations:** 1 Burn Institute, Department of Rehabilitation Medicine, Hangang Sacred Heart Hospital, College of Medicine, Hallym University, Seoul 07247, Republic of Korea; bioeast007@naver.com (H.S.C.); pjhlyr@naver.com (Y.R.L.); nym8060@hanmail.net (Y.M.R.); 2 Department of Rehabilitation Medicine, Hangang Sacred Heart Hospital, College of Medicine, Hallym University, Seoul 07247, Republic of Korea; anyany98@naver.com (S.Y.J.); hamays@hanmail.net (Y.S.C.); 3 Department of Anesthesiology and Pain Medicine, Hangang Sacred Heart Hospital, College of Medicine, Hallym University, Seoul 07247, Republic of Korea; 031132@hallym.or.kr; 4 Department of Plastic and Reconstructive Surgery, Hangang Sacred Heart Hospital, College of Medicine, Hallym University, Seoul 07247, Republic of Korea

**Keywords:** post-burn hypertrophic scar, fibroblast, exosomes, melanogenesis, melanocytes, melanin

## Abstract

Post-burn hypertrophic scars often exhibit abnormal pigmentation. Exosomes play important roles in maintaining normal physiological homeostasis and in the pathological development of diseases. This study investigated the effects of the exosomes derived from hypertrophic scar fibroblasts (HTSFs) on melanocytes, which are pigment-producing cells. Normal fibroblasts (NFs) and HTSFs were isolated and cultured from normal skin and hypertrophic scar (HTS) tissue. Both the NF- and HTSF-exosomes were isolated from a cell culture medium and purified using a column-based technique. The normal human epidermal melanocytes were treated with both exosomes at a concentration of 100 μg/mL at different times. The cell proliferation, melanin content in the medium, apoptotic factors, transcription factors, melanin synthesis enzymes, signaling, signal transduction pathways, and activators of transcription factors (STAT) 1, 3, 5, and 6 were investigated. Compared with the Dulbecco’s phosphate-buffered saline (DPBS)-treated controls and NF-exosomes, the HTSF-exosomes decreased the melanocyte proliferation and melanin secretion. The molecular patterns of apoptosis, proliferation, melanin synthesis, Smad and non-Smad signaling, and STATs were altered by the treatment with the HTSF-exosomes. No significant differences were observed between the DPBS-treated control and NF-exosome-treated cells. HTSF-derived exosomes may play a role in the pathological epidermal hypopigmentation observed in patients with HTS.

## 1. Introduction

Hypertrophic scars (HTSs) are a major negative outcome that often occurs in patients with thermal injuries. Approximately 70% of the patients experience deep second- or third-degree burn trauma [1]. It is frequently accompanied with itching, pain, and functional problems if a thick scar (contracture) occurs at the joint junction [1]. Additionally, the development of HTSs carries a high risk of hypopigmentation, which refers to the loss of skin color, resulting in lighter patches [2]. For HTSs, this can be aesthetically bothersome, especially if a scar is visible. Patients may feel self-conscious about the contrast between the hypopigmented scar and surrounding skin [3]. This can also affect the patient’s self-esteem and quality of life, leading to emotional distress, anxiety, and social withdrawal. Importantly, hypopigmentation indicates a lack of melanin in the area, which provides natural protection against ultraviolet (UV) radiation. The areas that are more sensitive to sunlight are more vulnerable to sunburn and other UV-related damage [2]. Hypopigmentation can also affect thermoregulation. Melanin plays an important role in heat absorption and dissipation. Scar tissue lacking melanin may not regulate the temperature effectively [2]. Currently, the treatment of hypopigmentation is challenging because there is no definitive therapy. The treatment options include laser treatment, topical agents, and surgical excision; however, the outcomes vary [3]. Therefore, it is necessary to clarify the pathogenesis of hypopigmentation in post-burn HTSs.

Dermal fibroblasts play an important role in the development of post-burn HTSs [4,5]. These cells proliferate and differentiate at the site of injury during the healing process, producing an extracellular matrix (ECM) that fills wounds and promotes wound healing. However, under pathological conditions, including prolonged inflammation, decreased apoptosis activity, and significantly increased levels of transforming growth factor-beta1 (TGF-β1) in the peripheral blood, the fibroblasts become overactivated and secrete excessive ECM, ultimately leading to HTS formation [Fs4, 7]. Therefore, fibroblasts are recognized as the cellular pathological basis of HTSs. Compared with normal fibroblasts (NFs), HTS fibroblasts (HTSFs) exhibit an altered pathological phenotype. For instance, the levels of TGF-β1, the myofibroblast marker alpha-smooth muscle actin (α-SMA), and ECM components such as fibronectin, collagen, and connective tissue growth factor (CTGF) are increased in HTSFs [5,6,7,8,9]. Additionally, the expression levels of inflammation-related toll-like receptor 4 (TLR-4) and interleukin 6 (IL-6) are elevated in HTSFs [10,11].

In the epidermis, the melanin pigment originates from the melanosomes in melanocytes, which are specialized melanin-producing cells, and its accumulation and distribution determine the skin color [12]. The biosynthetic process, which converts the amino acid tyrosine into melanin, is regulated by several enzymes. These include tyrosinase (TYR), tyrosinase-related protein 1 (TRP1), and dopachrome tautomerase, which is also known as tyrosinase-related protein 2 (TRP2) [13]. Among them, TYR is a rate-limiting enzyme [12,13]. Melanocyte proliferation and melanin synthesis are mainly regulated by adrenocorticotropic hormone (ACTH) and α-melanocyte stimulating hormone (α-MSH), both of which exhibit mitogenic and melanogenic activities [13]. UV radiation specifically triggers melanocytes to begin melanin synthesis. This is a protective response to shield the DNA in skin cells from UV-induced damage. UV radiation not only activates TYR but also stimulates the production of various signaling molecules and hormones, such as ACTH and α-MSH [14]. Additionally, growing studies have shown that numerous endogenous factors including endothelin, histamine, eicosanoids, catecholamines, estrogens, androgens, serotonin, corticosteroids, melatonin, dopamine, acetylcholine, melanin-concentrating hormone (MCH), and multiple cytokines also positively or negatively regulate melanogenesis [12].

Increasing studies have elucidated the functional role of fibroblast-derived paracrine factors including keratinocyte growth factor (KGF), TGF-β1, fibroblast activation protein-a (FAPα), Dickkopf-1, and corticotropin-releasing hormone (CRH) that regulate melanocyte proliferation or melanin synthesis [15,16,17,18,19]. These factors not only regulate the activity of TYR and TRP1 but also control the expression of several transcription factors, such as melanocyte-inducing transcription factor (MITF), SRY-related HMG-box-10 (Sox10), and paired box-3 (Pax3). Pax3 collaborates with Sox10 in controlling MITF expression, which plays a vital role in melanogenesis and pigmentary disorders [13,20].

Exosomes are small extracellular vesicles that are typically 30–100 nm in diameter and are produced within the endosomal compartments of most eukaryotic cells [20]. Exosomes carry their own genetic characteristics, including proteins, lipids, DNA, and RNAs, which are released from the cell into the extracellular space and bind to the receptors of recipient cells to regulate their physiological functions and participate in pathological processes [21]. Therefore, exosomes have been recognized as potential biomarkers. Importantly, they are involved in several fibrotic diseases, including liver [22], renal [23], and skin fibroses [24,25]. Recently, we observed that the exosomes derived from HTSFs exhibited profibrotic properties through the activation of Smad and transforming growth factor beta-activated kinase 1 (TAK1) signaling and increased expression of fibrosis markers with α-SMA, fibronectin, and collagen when treating NFs [24]. Moreover, exogenous treatment induces pathological changes in the proliferation and differentiation of normal keratinocytes [25].

Although melanocytes are surrounded by keratinocytes that form the epidermal melanin unit (EMU), they are located in the stratum basal layer, which is adjacent to the dermis layer [12]. Therefore, communication may occur between melanocytes and fibroblasts, the major cell types in the dermis. The development of post-burn HTSs is conventionally considered to be a dermal pathology. Therefore, the effects of dermal fibroblasts on melanocyte behavior cannot be ignored. Additionally, exosomes serve as a means of communication between cells. Therefore, we hypothesized that the exosomes derived from HTSFs may have pathological effects on melanocyte function. Generally, the crosstalk between fibroblasts and other epithelial cells (keratinocytes and melanocytes) occurs under normal physiological conditions in a conditioned culture medium. However, fibroblast growth requires serum supplementation, whereas epithelial cell growth is inhibited in the presence of serum. Exosomes can overcome this problem and provide a good tool for research on cell-to-cell communication. 

To the best of our knowledge, this is the first study to report the altered melanocyte activity and function after treatment with exosomes isolated from hypopigmented HTSFs. In this study, we investigated the effects of HTSF-exosomes on the molecular expression and signaling related to melanogenesis in normal human epidermal melanocytes (NHEMs). These results provide new insights into the pathological role of HTSF-exosomes in hypopigmentation during post-burn HTSs.

## 2. Results

### 2.1. HTSF-Exosomes Inhibit Proliferation and Production of Melanin

**Figure 1:**
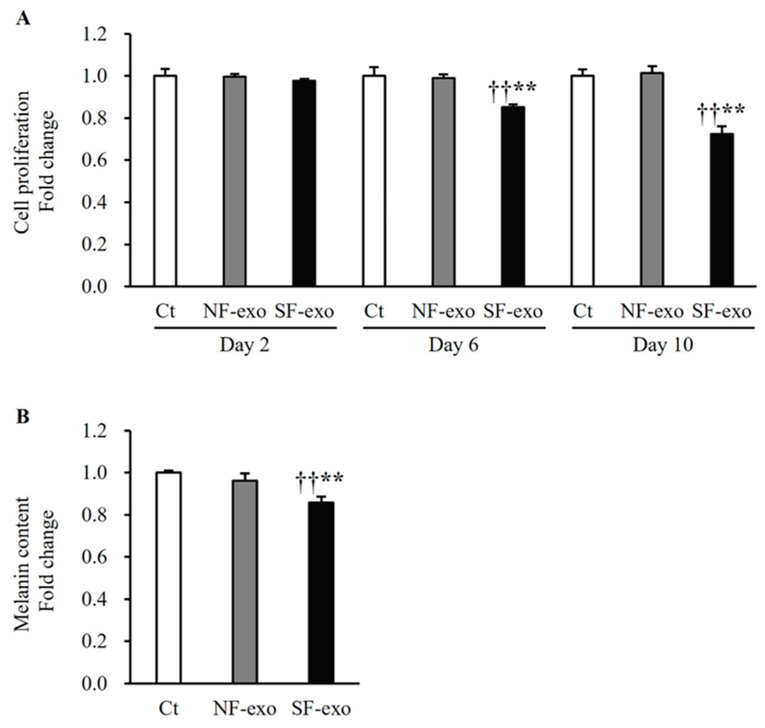
Effects of exosomes derived from NFs and SFs on proliferation and melanin production in NHEMs. (**A**) NHEMs were treated with Dulbecco’s phosphate-buffered saline (DPBS), NF-exo (100 μg/mL), and SF-exo (100 μg/mL) every 2 days for 2, 6, and 10 days, respectively. SF-exo treatment significantly decreased proliferation of NHEMs at 6 and 10 days compared with treatment with DPBS or NE-exo. (**B**) SF-exo (100 μg/mL) treatment for 2 days significantly decreased the melanin content in the cell culture medium compared with treatment with DPBS or NE-exo (100 μg/mL). Data are expressed as means ± standard deviations; n = 4 (each group). Ct, DPBS-treated cells; NHEMs, normal human epidermal melanocytes; NF-exo, normal fibroblast-derived exosomes; SF-exo, hypertrophic scar fibroblast-derived exosomes. ** *p* < 0.01, vs. Ct; †† *p* < 0.01, vs. NF-exo.

The NHEMs exposed to 100 μg exosomes derived from NFs (NF-exo) and HTSFs (SF-exo) for 2 days showed no significant change in proliferation compared with those treated with Dulbecco’s phosphate-buffered saline (DPBS) as the control (NF-exo vs. DPBS: 0.99 ± 0.01-fold and SF-exo vs. DPBS: 0.97 ± 0.01-fold; *p* > 0.05; Figure 1A). No significant difference in cell proliferation was observed between the treatments with NF-exo and DPBS on days 6 and 10 (NF-exo vs. DPBS: 0.98 ± 0.01 and 1.0 ± 0.03-fold, respectively; *p* > 0.05; Figure 1A). However, the treatment with SF-exo for 6 and 10 days significantly inhibited the proliferation of the NHEMs compared with the treatment with DPBS or NF-exo (SF-exo vs. DPBS: 0.85 ± 0.01 and 0.72 ± 0.04-fold, SF-exo vs. NF-exo: 0.86 ± 0.02 and 0.71 ± 0.03-fold, respectively; *p* < 0.01; Figure 1A). The treatment with SF-exo for 2 days significantly decreased the melanin production of the NHEMs compared with the treatment with DPBS or NF-exo; this was observed by measuring the melanin content in the medium (SF-exo vs. DPBS: 0.85 ± 0.01-fold; *p* < 0.01; Figure 1B). The NF-exo treatment did not influence the melanin production compared with the DPBS treatment (NF-exo vs. DPS: 0.96 ± 0.03-fold; *p* > 0.05; Figure 1B). SF-exo suppressed melanogenesis by inhibiting cell growth and melanin production. 

### 2.2. HTSF-Exosomes Did Not Induce Apoptosis

**Figure 2:**
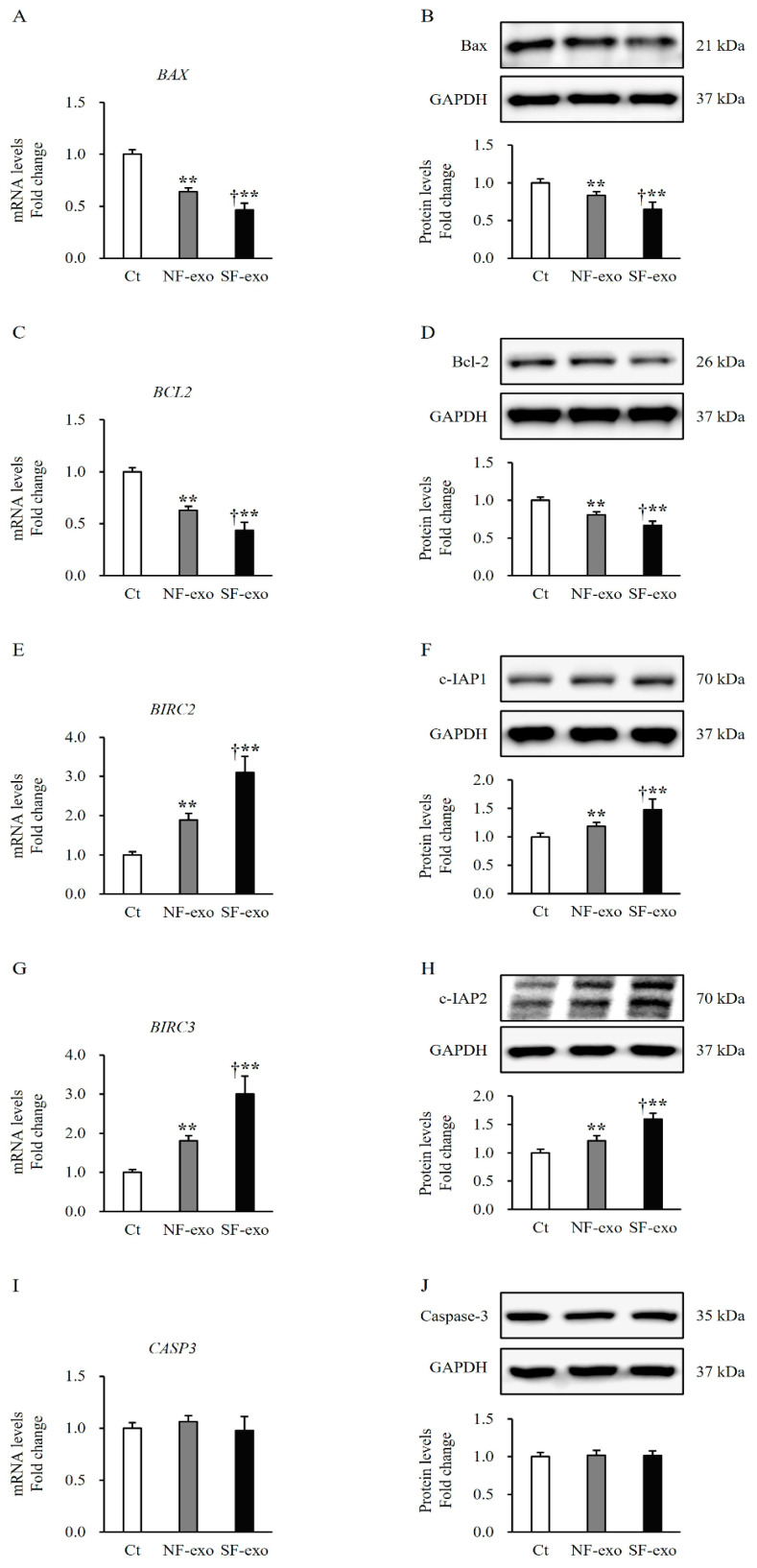
Effects of exosomes derived from NFs and SFs on apoptosis of NHEMs. NHEMs were treated with DPBS, NF-exo (100 μg/mL), and SF-exo (100 μg/mL) for 2 days. Significantly decreased mRNA and protein expressions of bax (**A**,**B**) and bcl2 (**C**,**D**) in NHEMs treated with NF-exo or SF-exo compared with those in DPBS-treated cells. Significantly decreased mRNA and protein expressions of bax (**A**,**B**) and bcl2 (**C**,**D**) in NHEMs treated with SF-exo compared with those in NF-exo-treated cells. Significantly increased mRNA and protein expressions of c-IAP1 (**E**,**F**) and c-IAP2 (**G**,**H**) in NHEMs treated with NF-exo or SF-exo compared to those in DPBS-treated cells. Significantly increased mRNA and protein expressions of c-IAP1 (**E**,**F**) and c-IAP2 (**G**,**H**) in NHEMs treated with SF-exo compared to those in NF-exo-treated cells. No difference in mRNA and protein expressions of caspase 3 (**I**,**J**) in NHEMs treated with SF-exo or NF-exo compared to those in DPBS-treated cells. Data are expressed as the mean ± standard deviations; n = 3 (each group). Ct, DPBS treated cells; NHEMs, normal human epidermal melanocytes; NF-exo, normal fibroblast-derived exosomes; SF-exo, hypertrophic scar fibroblast-derived exosomes; GADPH, glyceraldehyde 3-phosphate dehydrogenase. † *p* < 0.05, vs. NF-exo; ** *p* < 0.01, vs. Ct.

To investigate the effects of HTSF-exosomes on apoptosis in NHEMs, the expression of several apoptosis-related molecules was analyzed using quantitative reverse transcription polymerase chain reaction (qRT-PCR) and Western blotting. The treatment with 100 μg/mL of SF-exo or NF-exo for 2 days significantly decreased the mRNA and protein expressions of Bax, a pro-apoptotic marker (SF-exo vs. DPBS: mRNA, 0.46 ± 0.07-fold and protein, 0.63 ± 0.09-fold; NF-exo vs. DPBS: mRNA, 0.64 ± 0.04-fold and protein, 0.83 ± 0.05-fold; *p* < 0.01; Figure 2A), and Bcl2, an anti-apoptotic marker (SF-exo vs. DPBS: mRNA, 0.44 ± 0.08-fold and protein, 0.66 ± 0.05-fold; NF-exo vs. DPBS: mRNA, 0.63 ± 0.04-fold and protein, 0.80 ± 0.04-fold; *p* < 0.01; Figure 2B), compared with the treatment with DPBS in NHEMs. Moreover, both the mRNA and protein expression levels of Bax and Bcl2 in the NHEMs treated with SF-exo were lower than those in the NHEMs treated with NF-exo (Bax, SF-exo vs. NF-exo: mRNA, 0.71 ± 0.06-fold and protein, 0.77 ± 0.06-fold; Bcl2, SF-exo vs. NF-exo: mRNA, 0.70 ± 0.05-fold and protein, 0.81 ± 0.04-fold, *p* < 0.05; Figure 2A,B). However, the treatment with SF-exo or NF-exo significantly increased the mRNA and protein expressions of inhibitors of apoptosis 1 (c-IAP1) (SF-exo vs. DPBS: mRNA, 3.10 ± 0.41-fold and protein, 1.48 ± 0.19-fold; NF-exo vs. DPBS: mRNA, 1.88 ± 0.16-fold and protein, 1.18 ± 0.07-fold; *p* < 0.01; Figure 2C) and c-IAP2 (SF-exo vs. DPBS: mRNA, 3.10 ± 0.41-fold and protein, 1.48 ± 0.19-fold; NF-exo vs. DPBS: mRNA, 1.81 ± 0.12-fold and protein, 1.21 ± 0.08-fold; *p* < 0.01; Figure 2D), which are anti-apoptotic markers in NHEMs. Moreover, both the mRNA and protein expression levels of c-IAP1 and c-IAP2 in the NHEMs treated with SF-exo were higher than those in the NHEMs treated with NF-exo (c-IAP1, SF-exo vs. NF-exo: mRNA, 1.64 ± 0.10-fold and protein, 1.25 ± 0.05-fold; c-IAP2, SF-exo vs. NF-exo: mRNA, 1.65 ± 0.11-fold and protein, 1.31 ± 0.07-fold; *p* < 0.05; Figure 2C,D). The mRNA and protein expressions of caspase 3, which are hallmarks of apoptosis, were not changed by the treatment with SF-exo or NF-exo compared with the treatment with DPBS (SF-exo vs. DPBS: mRNA, 1.06 ± 0.06-fold and protein, 1.02 ± 0.08-fold; NF-exo vs. DPBS: mRNA, 1.03 ± 0.04-fold and protein, 1.05 ± 0.07-fold; *p* > 0.05; Figure 2E). These results suggested that SF-exo neither induces apoptosis in NHEMs nor inhibits melanogenesis, which is not attributed to cell apoptosis. 

### 2.3. HTSF-Exosomes Decreased the Expression of Melanogenesis-Related Transcription Factors

**Figure 3:**
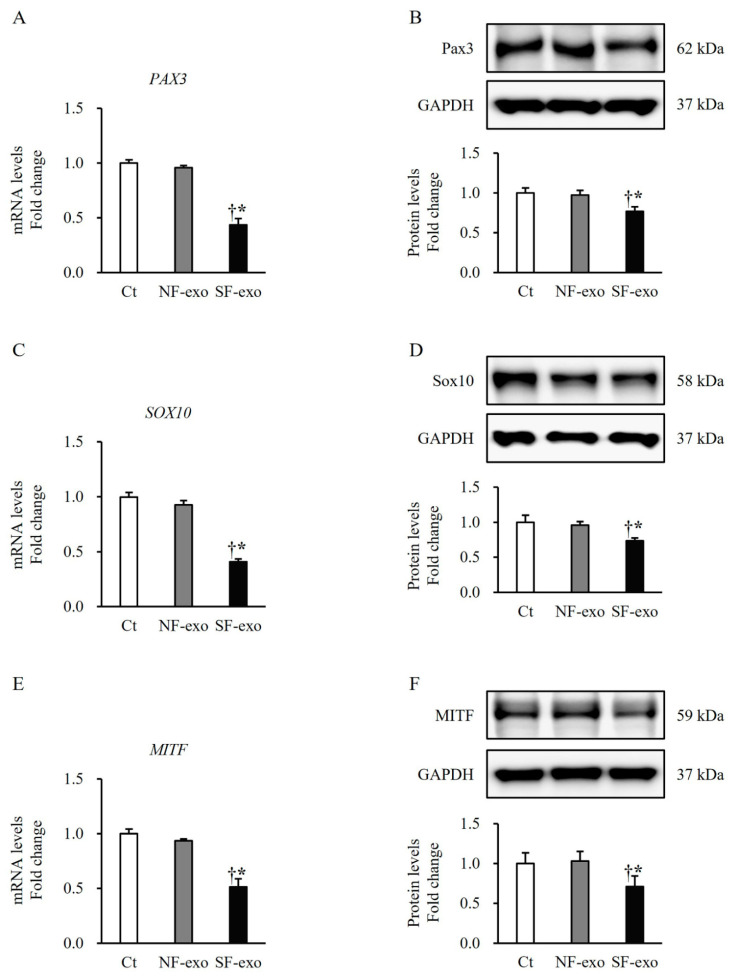
Effect of exosomes derived from NFs and SFs on expression of transcription factors involved in melanogenesis. NHEMs were treated with DPBS, NF-exo (100 μg/mL), and SF-exo (100 μg/mL) for 2 days. Significantly decreased mRNA and protein expressions of Pax3 (**A**,**B**), Sox10 (**C**,**D**), and MITF (**E**,**F**) in NHEMs treated with SF-exo for 2 days compared with those in DPBS- or NF-exo-treated cells. Data are expressed as means ± standard deviations; n = 3 (each group). Ct, DPBS-treated cells; NHEMs, normal human epidermal melanocytes; NF-exo, normal fibroblast-derived exosomes; SF-exo, hypertrophic scar fibroblast-derived exosomes; GADPH, glyceraldehyde 3-phosphate dehydrogenase. † *p* < 0.05, vs. NF-exo; * *p* < 0.01, vs. Ct.

To elucidate the inhibitory effects of SF-exo on the melanogenesis in NHEMs, transcription factor expression was evaluated. The NHEMs treated with SF-exo showed significantly decreased mRNA and protein expressions of Pax 3 (SF-exo vs. DPBS: mRNA, 0.44 ± 0.06-fold and protein, 0.76 ± 0.06-fold; SF-exo vs. NF-exo: mRNA, 0.44 ± 0.06-fold and protein, 0.78 ± 0.03-fold; *p* < 0.05; Figure 3A,B), Sox 10 (SF-exo vs. DPBS: mRNA, 0.41 ± 0.03-fold and protein, 0.73 ± 0.04-fold; SF-exo vs. NF-exo: mRNA, 0.45 ± 0.07-fold and protein, 0.76 ± 0.04-fold; *p* < 0.05; Figure 3C,D), and MITF (SF-exo vs. DPBS: mRNA, 0.51 ± 0.07-fold and protein, 0.70 ± 0.11-fold; SF-exo vs. NF-exo: mRNA, 0.54 ± 0.07-fold and protein, 0.68 ± 0.05-fold; *p* < 0.05; Figure 3E,F) compared with those treated with DPBS or NF-exo. However, the NHEMs treated with NF-exo did not show changes in the mRNA and protein expressions of Pax 3 (NF-exo vs. DPBS: mRNA, 0.96 ± 0.06-fold and protein, 0.97 ± 0.06-fold; *p* > 0.05; Figure 3A,B), Sox 10 (NF-exo vs. DPBS: mRNA, 0.93 ± 0.04-fold and protein, 0.96 ± 0.05-fold; *p* > 0.05; Figure 3C,D), and MITF (NF-exo vs. DPBS: mRNA, 0.95 ± 0.02-fold and protein, 1.01 ± 0.09-fold; *p* > 0.05; Figure 3E,F) compared with those treated with DPBS. These results suggest that SF-exo suppresses melanogenesis by downregulating the expression of melanogenesis-related transcription factors. 

### 2.4. HTSF-Exosomes Decreased the Expression of Melanin Synthesis-Related Regulatory Enzymes

**Figure 4:**
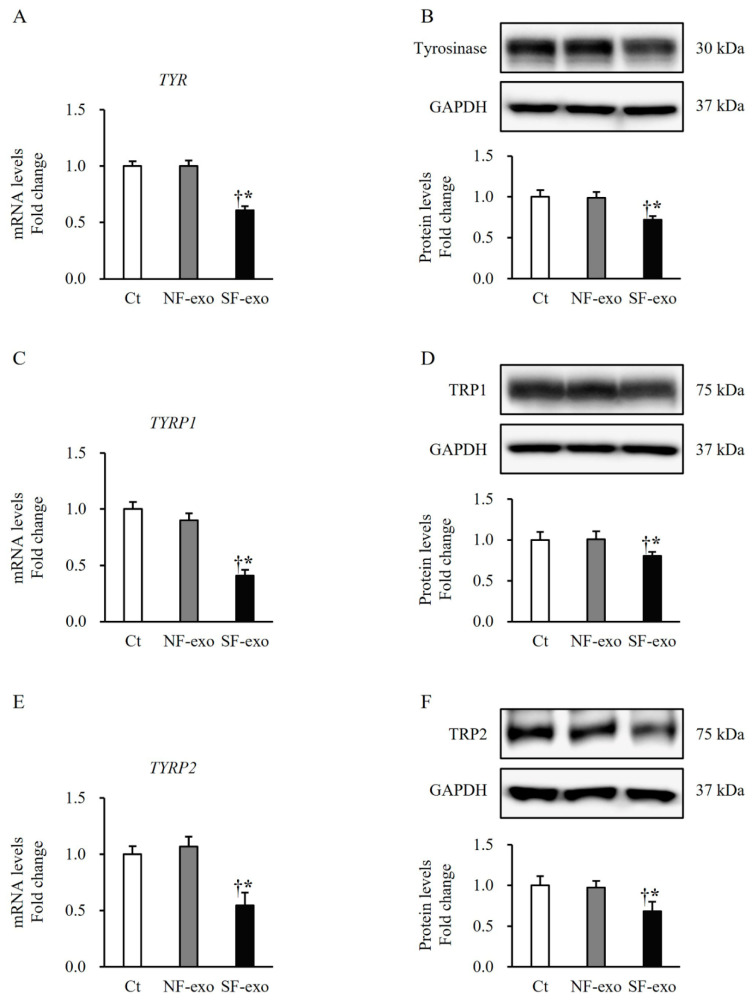
Effects of exosomes derived from NFs and SFs on the expression of melanin synthesis-related enzymes in NHEMs. NHEMs were treated with DPBS, NF-exo (100 μg/mL), and SF-exo (100 μg/mL) for 2 days. mRNA and protein expressions of tyrosinase (**A**,**B**), TRP1 (**C**,**D**), and TRP2 (**E**,**F**) in NHEMs treated with SF-exo were significantly decreased compared with those in DPBS- or NF-exo-treated cells. Data are expressed as means ± standard deviations; n = 3 (each group). Ct, DPBS-treated cells; NHEMs, normal human epidermal melanocytes; NF-exo, normal fibroblast-derived exosomes; SF-exo, hypertrophic scar fibroblast-derived exosomes; TRP, tyrosinase-related protein; GADPH, GADPH, glyceraldehyde 3-phosphate dehydrogenase. † *p* < 0.05, vs. NF-exo; * *p* < 0.05, vs. Ct.

To elucidate the inhibitory effects of SF-exo on the melanogenesis in NHEMs, we evaluated the expression of the enzymes that regulate melanin biosynthesis. The NHEMs treated with SF-exo showed significantly decreased mRNA and protein expressions of tyrosinase (SF-exo vs. DPBS: mRNA, 0.61 ± 0.04-fold and protein, 0.71 ± 0.05-fold; SF-exo vs. NF-exo: mRNA, 0.60 ± 0.05-fold and protein, 0.72 ± 0.04-fold; *p* < 0.05; Figure 4A*,*B), TRP1 (SF-exo vs. DPBS: mRNA, 0.41 ± 0.05-fold and protein, 0.79 ± 0.05-fold; SF-exo vs. NF-exo: mRNA, 0.46 ± 0.07-fold and protein, 0.79 ± 0.05-fold; *p* < 0.05; Figure 4C*,*D), and TRP2 (SF-exo vs. DPBS: mRNA, 0.54 ± 0.10-fold and protein, 0.68 ± 0.11-fold; SF-exo vs. NF-exo: mRNA, 0.51 ± 0.06-fold and protein, 0.70 ± 0.04-fold; *p* < 0.05; Figure 4E*,*F) compared with those treated with DPBS or NF-exo. However, the NHEMs treated with NF-exo did not exhibit changes in the mRNA and protein expressions of tyrosinase (NF-exo vs. DPBS: mRNA, 1.0 ± 0.05-fold and protein, 0.99 ± 0.07-fold; *p* > 0.05; Figure 4A*,*B), TRP1 (NF-exo vs. DPBS: mRNA, 0.90 ± 0.06-fold and protein, 1.0 ± 0.08-fold; *p* > 0.05; Figure 4C*,*D), and TRP2 (NF-exo vs. DPBS: mRNA, 1.03 ± 0.08-fold and protein, 0.97 ± 0.05-fold; *p* > 0.05; Figure 4E*,*F) compared with those treated with DPBS. SF-exo partially suppressed melanogenesis by decreasing the expression of the enzymes involved in melanin synthesis.

### 2.5. HTSF-Exosomes Induce Melanogenesis-Related Signaling Changes

**Figure 5:**
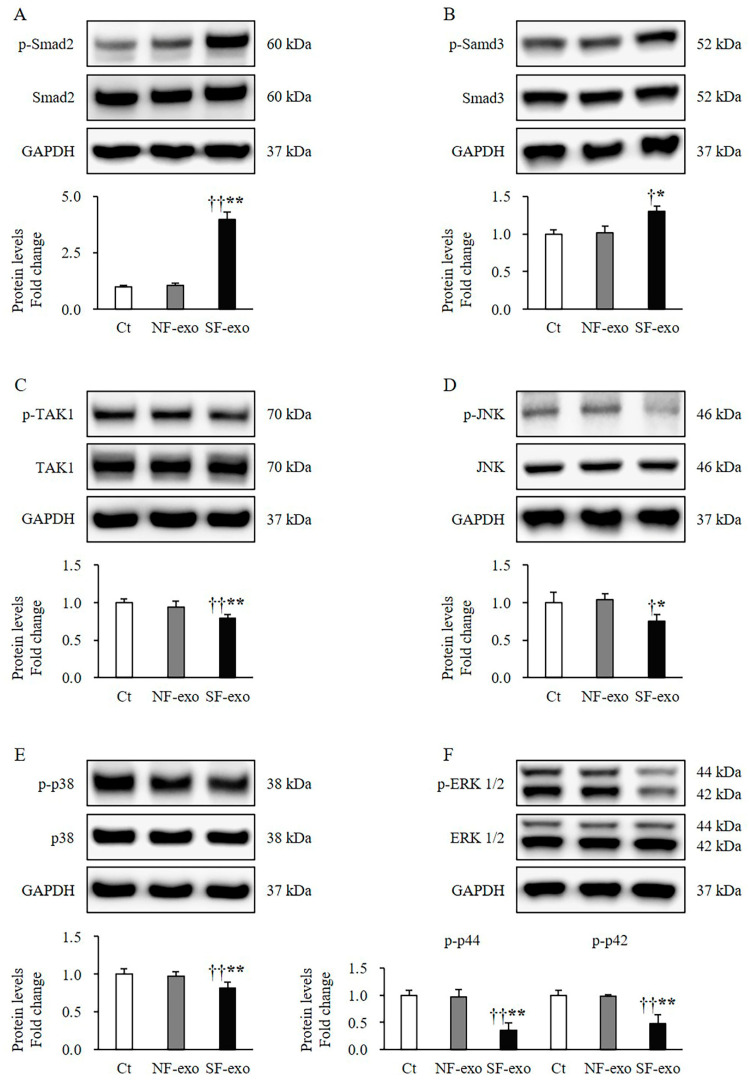
Effects of exosomes derived from NFs and SFs on Smad-dependent and -independent signaling in NHEMs. NHEMs were treated with DPBS, NF-exo (100 μg/mL), and SF-exo (100 μg/mL) for 2 days. Significantly increased phosphorylation levels of Smad2 (**A**) and Smad3 (**B**) in NHEMs treated with SF-exo compared with those in NF-exo or DPBS-treated cells. Significantly decreased phosphorylation levels of TAK1 (**C**), JNK (**D**), p38 (**E**), and ERK1/2 (**F**) in NHEMs treated with SF-exo compared with those in DPBS- or NF-exo-treated cells. Data are expressed as means ± standard deviations; n = 3 (each group). Ct, DPBS-treated cells; NHEMs, normal human epidermal melanocytes; NF-exo, normal fibroblast-derived exosomes; SF-exo, hypertrophic scar fibroblast-derived exosomes. † *p* < 0.05 or †† *p* < 0.01, vs. NF-exo; * *p* < 0.05 or ** *p* < 0.01, vs. Ct.

The NHEMs treated with SF-exo for 2 days showed significantly increased phosphorylation levels of Smad2 (SF-exo vs. DPBS: 3.98 ± 0.10-fold; SF-exo vs. NF-exo: 3.72 ± 0.11-fold; *p* < 0.01; Figure 5A) and Smad3 (SF-exo vs. DPBS: 1.30 ± 0.06-fold; SF-exo vs. NF-exo: 1.29 ± 0.08-fold; *p* < 0.05; Figure 5B) compared with those treated with DPBS or NF-exo. Moreover, the phosphorylation of TAK1 (SF-exo vs. DPBS: 0.76 ± 0.05-fold; SF-exo vs. NF-exo: 0.77 ± 0.05-fold; *p* < 0.01; Figure 5C), c-Jun N-terminal kinase (JNK) (SF-exo vs. DPBS: 0.75 ± 0.08-fold; SF-exo vs. NF-exo: 1.29 ± 0.07-fold; *p* < 0.05; Figure 5D), p38 (SF-exo vs. DPBS: 0.75 ± 0.05-fold; SF-exo vs. NF-exo: 0.79 ± 0.04-fold; *p* < 0.01; Figure 5E), and ERK1/2 (SF-exo vs. DPBS: ERK1, 0.35 ± 0.14-fold and ERK2, 0.47 ± 0.16-fold; SF-exo vs. NF-exo: ERK1, 0.36 ± 0.13-fold and ERK2, 0.47 ± 0.15-fold; *p* < 0.01; Figure 5F) was significantly decreased with the treatment with SF-exo compared with the treatment with DPBS or NF-exo. However, the treatment with NF-exo for 2 days did not affect the phosphorylation of Smad2 (NF-exo vs. DPBS: 1.07 ± 0.08-fold; *p* > 0.05; Figure 5A), Smad3 (NF-exo vs. DPBS: 1.01 ± 0.06-fold; *p* > 0.05; Figure 5B), TAK1 (NF-exo vs. DPBS: 0.94 ± 0.07-fold; *p* > 0.05; Figure 5C), JNK (NF-exo vs. DPBS: 1.12 ± 0.09-fold; *p* > 0.05; Figure 5D), p38 (NF-exo vs. DPBS: 0.86 ± 0.11-fold; *p* > 0.05; Figure 5E), or ERK1/2 (NF-exo vs. DPBS: ERK1, 0.97 ± 0.09-fold and ERK2, 0.99 ± 0.08-fold; *p* > 0.05; Figure 5F) compared with the treatment with DPBS in NHEMs. Accordingly, the SF-exo treatment caused changes in Smad and non-Smad signaling.

### 2.6. HTSF-Exosomes Increase Phosphorylation of Transcription Factors 

**Figure 6:**
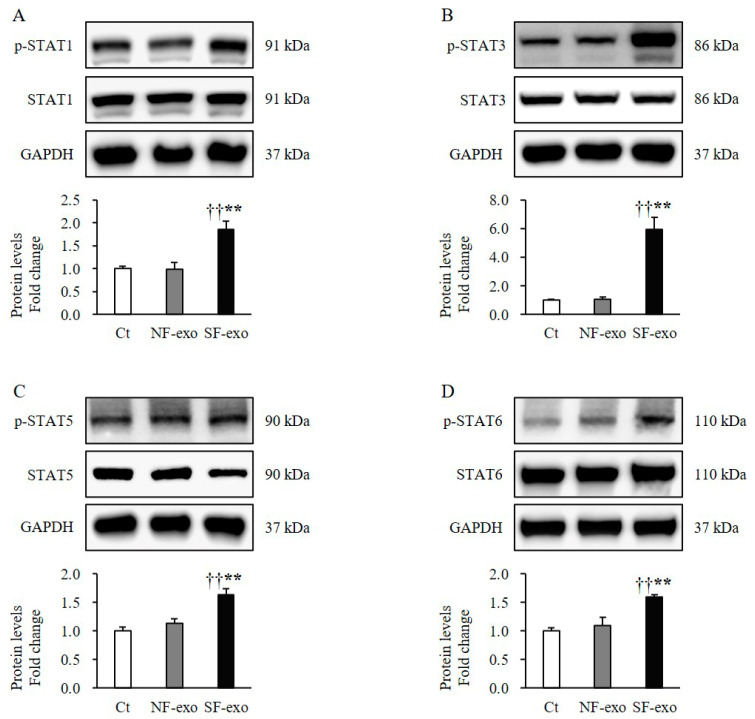
Effects of exosomes derived from NFs and SFs on phosphorylation of transcription factors in NHEMs. NHEMs were treated with DPBS, NF-exo (100 μg/mL), and SF-exo (100 μg/mL) for 2 days. Phosphorylation levels of STAT1 (**A**), STAT3 (**B**), STAT5 (**C**), and STAT6 (**D**) in NHEMs treated with SF-exo significantly increased compared with those in NF-exo or DPBS-treated cells. Data are expressed as means ± standard deviations; n = 4 (each group). Ct, DPBS-treated cells; NHEMs, normal human epidermal melanocytes; NF-exo, normal fibroblast-derived exosomes; SF-exo, hypertrophic scar fibroblast-derived exosomes. †† *p* < 0.01, vs. NF-exo; ** *p* < 0.01, vs. Ct.

The NHEMs treated with SF-exo for 2 days showed significantly increased phosphorylation levels of the signal transducers and activators of transcription factor (STAT)1 (SF-exo vs. DPBS: 1.86 ± 0.16-fold; SF-exo vs. NF-exo: 1.89 ± 0.11-fold; *p* < 0.01; Figure 6A), STAT3 (SF-exo vs. DPBS: 5.82 ± 0.87-fold; SF-exo vs. NF-exo: 5.44 ± 0.73-fold; *p* < 0.01; Figure 6B), STAT5 (SF-exo vs. DPBS: 1.63 ± 0.11-fold; SF-exo vs. NF-exo: 1.46 ± 0.09-fold; *p* < 0.01; Figure 6C), and STAT6 (SF-exo vs. DPBS: 1.59 ± 0.04-fold; SF-exo vs. NF-exo: 1.46 ± 0.08-fold; *p* < 0.01; Figure 5D) compared with those treated with DPBS or NF-exo. However, the treatment with NF-exo for 2 days did not affect the phosphorylation of STAT1 (NF-exo vs. DPBS: 0.98 ± 0.15-fold; *p* > 0.05; Figure 5A), STAT3 (NF-exo vs. DPBS: 1.07 ± 0.14-fold; *p* > 0.05; Figure 5B), STAT5 (NF-exo vs. DPBS: 1.13 ± 0.15-fold; *p* > 0.05; Figure 5C), or STAT6 (NF-exo vs. DPBS: 1.09 ± 0.15-fold; *p* > 0.05; Figure 5D) compared with the treatment with DPBS in NHEMs. SF-exo suppressed melanogenesis by activating STAT1, 3, 5, and 6.

### 2.7. Expression of miRNAs in HTSFs-Exosomes

The microarray results revealed that the expression levels of the 12 miRNAs in the SF-exo-treated NHEMs were markedly higher than those in the NF-exo-treated NHEMs (Table 1). In particular, these miRNAs are known to be related to the suppression of melanogenesis, including Hsa-let-7a-2-3p (10.2-fold increase), Hsa-let-7b-3p (20.3-fold increase), Hsa-let-7e-3p (243.3-fold increase), Hsa-let-7f-1-3p (33.7-fold increase), Hsa-let-7i-3p (13.4-fold increase), Hsa-miR-31-5p (6.0-fold increase), Hsa-miR-342-3p (8.8-fold increase), Hsa-miR-125a-5p (11.5-fold increase), Hsa-miR-365a-5p (73.9-fold increase), Hsa-miR-125b-5p (5.0-fold increase), Hsa-miR-138-5p (3.5-fold increase), and Hsa-miR-605-5p (64.9-fold increase) (Table 1). SF-exo partially suppressed melanogenesis by upregulating the miRNAs. 

## 3. Discussion

An increasing number of studies have focused on the pathological role of exosomes in the development and progression of multiple-organ diseases. The exosomes released by pathological cells can act as efficient messengers in cell-to-cell communication locally (toward recipient cells) or systemically (via circulation), inducing the transformation of healthy cells into pathological phenotypes. The exosomes derived from the bronchoalveolar lavage fluid (BALF) of patients promote lung diseases including chronic inflammation, fibrosis, and cancer. Exosomes are composed of proteins, cytokines, and others [26]. The miR-30a-5p expression was downregulated in BALF-exosomes, and in vitro studies have revealed that the overexpression of miR-30a-5p reduces the TAK1 signaling, α-SMA, and fibronectin levels in A549 cells [27]. Abundant TGF-β1-containing exosomes were secreted from kidney tubular epithelial cells under hypoxic conditions, and the exosomes increased the cell proliferation and the expression of fibrosis markers α-SMA and type I collagen in renal fibroblasts. However, exosomes following the abolished transcription of TGF-β1 mRNA, increasing the expression of the abovementioned fibrosis markers, were not observed [23]. Moreover, different types of stem cell-derived exosomes hold significant promise as cell-free therapies in regenerative medicine, offering the benefits of stem cells without any associated risks. In our previous study, the SF-exosomes induced fibroblast–mesenchymal transition with increased expressions of N-cadherin and vimentin and differentiation with increasing α-SMA expression and synthesis of ECM when treating NFs [24]. These results suggest that SF-exosomes have a profibrotic property, like TGF-β1, and participate in HTS development. 

HTSs commonly occur after burn injuries and are characterized by a raised and thickened appearance. These scars may exhibit variations in pigmentation ranging from hyperpigmentation to hypopigmentation. In HTS hypopigmentation, a study using a Duroc pig dyschromia model showed that both the hyperpigmented and hypopigmented regions within HTSs had equal numbers of melanocytes. In addition, the number of cells in the dyspigmented regions was not different from that in the normal pigmented skin [28]. Thus, the differences in pigmentation may be caused by factors other than the melanocyte number. Animal research has indicated that the levels of ACTH, α-MSH, and their receptor (melanocortin 1 receptor) were upregulated in the hyperpigmented regions in comparison with hypopigmented scars [28]. Both ACTH and α-MSH induce melanogenesis by controlling the expression of transcription factors MITF, Sox10, and Pax3, which are critical for melanin synthesis enzymes TYR, TRP1, and TRP2 [13]. Upon further exploration, the MITF levels did not differ from those in the hypopigmented samples, although the TYR, TRP1, and TYP2 expression levels increased in the hyperpigmented samples [28]. However, contrasting findings were found from the immunohistochemistry analysis of hypopigmented human post-burn HTS tissues compared with normal skin [29]. The study results suggest that hypopigmentation in HTSs is associated with a reduced number of dendrites and melanocytes. Moreover, although a few melanocytes are present, the study did not sufficiently synthesize or transfer melanin to the keratinocytes, which are primarily cultured from both hypopigmented HTS and normal skin. However, the pathophysiological mechanisms underlying post-burn HTS hypopigmentation remain unclear.

Melanogenesis is the process by which melanocytes produce melanin. Apoptosis leads to decreased cell growth and melanin synthesis [30]. In the present study, the cell proliferation and melanin production decreased in the NHEMs treated with SF-exosomes, as shown in Figure 1. However, the NHEMs did not undergo apoptosis after the SF-exosome treatment, as shown in Figure 2. Decreased Bax expression was accompanied with decreased expression of Bcl2. Importantly, the expressions of c-IAP1 and 2 were increased, and both have anti-apoptotic properties that protect cells from apoptosis [31]; therefore, the expression of caspase 3 may be unaffected by SF-exosomes. Accordingly, the decreased cell growth and melanin synthesis are not attributed to apoptosis.

Sox10, Pax3, and MITF are transcription factors that form a regulatory network essential for the cell growth and melanin synthesis in human melanocytes. Sox10 activates the MITF, which in turn controls the cell proliferation as well as several genes critical for melanogenesis, including TYR, TYP1, and TYP2 [13,32]. Sox10 promotes melanocyte proliferation by activating minichromosome maintenance complex component 5 (MCM5) [33]. In the absence of Sox10, the MITF did not induce the expression of TYR in mature mouse melanocytes [32]. 

Pax3 is involved in the early stages of melanocyte development. Pax3, along with the transcription factor Hairy and enhancer of split 1 (HES-1) and the proliferation marker antigen Kiel 67 (Ki-67), are co-expressed in the melanocytes of normal human skin, indicating a less differentiated proliferative phenotype. Thus, Pax3 may help to maintain a population of proliferative melanocytes that respond to environmental stimuli [34,35]. Importantly, Pax3 interacts with Sox10 to induce MITF expression, whereas cyclic AMP-responsive element-binding proteins, as cofactors, contribute to cell proliferation [36]. Interestingly, the mutations in Sox10 or Pax3 failed to trans-activate the MITF promoter, further supporting the hypothesis that these two genes work together to regulate MITF expression [37]. Moreover, silencing Pax3 in melanocytes reduces the expression of both TRP2 and cyclin A2 (CCNA2), which are proliferation genes [38].

The MITF acts as a master regulator of the proliferation, differentiation, survival, and pigmentation of melanocytes. Previous studies have elucidated the mechanisms through which the MITF regulates the proliferation and melanin synthesis in melanocytes. The MITF positively regulates diaphanous homolog 1 (DIAPH1, DIA1) and controls p27Kip1-dependent G1 arrest, thereby promoting melanocyte proliferation [39]. The MITF serves as an oncogene and is expressed in approximately 80% of human melanomas [40]. The MITF knockdown in melanoma cells results in growth arrest [41], and the mutation of MITF-M, the main isoform of the MITF in melanocytes, reduces the melanocyte numbers, leading to a white color in mice [42]. The crosstalk between the MITF and the transcription factor EB regulates the expression of various genes involved in melanocyte proliferation and melanin synthesis [43,44]. The MITF also positively regulates the expression of Bcl2, an anti-apoptotic factor, in normal melanocytes and human cutaneous melanoma cells [45]. In contrast, the MITF exerts an anti-proliferative effect by activating p21Cip1 in melanoma cells [46]. Accordingly, the roles of Sox10, Pax3, and the MITF in melanocyte growth and melanin synthesis support our finding that both the proliferation of the NHEMs and melanin content in the culture medium decreased (Figure 1). Moreover, after the SF-exosome treatment, there was a decrease in the expression of Sox10, Pax3, MITF, TYR, TRP1, and TRP2 in the NHEMs, as depicted in Figure 3 and Figure 4.

A previous study reported that SF-exosomes have a stronger profibrotic ability to induce Smad and non-Smad signaling in normal dermal fibroblasts [24]. Another report indicated that the profibrotic cytokine TGF-β1 inhibits human primary melanocytes growth and melanin synthesis through inducing the phosphorylation of Smad2, promoting Smad2/Smad4 complex translocation to the nucleus, and then downregulating Pax3 expression [16]. TAK1 signaling is a part of the non-Smad signaling in the TGF-β1 signaling [47]. Therefore, it plays a significant role in HTS formation. The activation of TAK1 can induce the phosphorylation of NF-κB and mitogen-activated protein kinases, including JNK, extracellular signal-regulated kinase (ERK42/44), and p38 [48]. The TAK1 expression was upregulated in melanoma cells compared to NHEMs. Furthermore, the ectopic expression of miR-377 in melanoma cells reduced the TAK1/NF-κB signaling pathway, followed by a decrease in proliferation [49]. Thus, TAK1 signaling may play a functional role in melanocytes, although its physiological role in normal melanocytes remains unknown. The TAK1 downstream signaling pathways JNK, ERK, and p38 are all involved in the response to environmental stresses and exogenous stimuli, such as UV radiation and α-MSH. Their roles in melanogenesis, including melanocyte proliferation and synthesis, have been extensively studied. MITF activation is positively regulated by the phosphorylation of JNK, ERK, and p38 [50]. In our study, the Smad (Smad2 and Smad3) and non-Smad (TAK1, JNK, ERK, and p38) signaling were suppressed in the NHEMs treated with SF-exosomes (Figure 5). These results, combined with their role in melanogenesis, suggest that SF-exosomes inhibit the melanogenesis in NHEMs by suppressing Smad/non-Smad-TIMF signaling. 

Several studies on the effects of cytokines on pigmentation have indicated the involvement of STAT phosphorylation in melanogenesis. Interferon-γ (IFN-γ) is abundantly expressed in the skin lesions and plasma of patients with vitiligo, a chronic autoimmune disorder that causes patches of skin to lose pigment [51]. Moreover, the inhibitory effect of IFN-γ on the suppression of melanogenesis is achieved by the activation of STAT1 and 3. The effects of IFN-γ on the downregulation of melanogenesis were also confirmed in B16F10 melanoma cells [52]. IL-4 also increased the phosphorylation of STAT3 and 6 in NHEMs. TGF-β1, which inhibits melanogenesis, can also easily induce the phosphorylation of STAT3 via promoting Smad3 activation [15,53]. Furthermore, both IFN-γ and IL-4 demonstrated decreased expressions of the MITF, TYR, TRP1, and TRP2 in NHEMs [54]. Both IL-4 and TGF-β1 were highly expressed in HTS tissues and HTS fibroblasts compared with those in the controls [5,55]. Moreover, both are recognized as the molecular basis of HTS formation [4]. The phosphorylation of STAT5 was induced by the epidermal growth factor, although the cell growth increases in NHEMs [56]. In melanoma, the phosphorylation of STAT5 has been detected, and this was correlated with the expression of Bcl-XL, an antiapoptotic factor. Therefore, STAT5 activation protects cells from apoptosis and regulates melanocyte proliferation and the survival of melanocytes [57]. Altogether, these studies support our results that the phosphorylation of STAT1, 3, and 6 decreases the melanogenesis in the NHEMs treated with SF-exosomes (Figure 6).

A previous study has reported that miRNAs negatively regulate melanocyte proliferation and melanin synthesis. Specifically, overexpression or exogenous treatment has been shown to decrease the expression of MITF, TYR, TYP1, and TYP2 and reduce the melanin production in melanoma cells or melanocytes [58]. Our findings revealed that 12 miRNAs were highly upregulated in SF-exosomes compared to those in NF-exosomes (Table 1). They repress the MITF expression, reduce the cell growth, and inhibit the TYR activity in human melanoma cells [59]. Moreover, five of the twelve miRNAs belong to the let-7 family, which comprises tumor suppressor miRNAs that can negatively regulate cancer stem-like cells [60]. The suppressive effect of SF-exosomes on melanogenesis may be partially attributed to the high levels of these miRNAs. However, the exact roles and mechanisms of these 12 miRNAs in human melanocytes have not yet been reported, and we plan to investigate these in future studies.

The pathology of post-burn HTS hypopigmentation has not yet been fully explored. The results of this study clearly demonstrate that HTSF-exosomes contribute to scar hypopigmentation. However, given the distribution of the melanocytes in skin tissue and the crucial role of keratinocytes in melanogenesis, the impact of keratinocyte exosomes on melanocytes should not be overlooked. Therefore, in future studies, we plan to investigate the effects of the exosomes from the pathological keratinocytes of HTSs on melanogenesis.

## 4. Materials and Methods

### 4.1. Primary Fibroblast Culture

The Institutional Review Board of the Hallym University Hangang Sacred Heart Hospital Institutional Review Board (HG2023-012) approved the study protocol. Hypopigmented HTS tissues of patients injured with thermal burns were obtained from surgical procedures that were performed 1–2 years after injury, and normal skin was derived by skin biopsies, which were site-matched with HTS tissues. The demographic and burn injury characteristics of the patients who developed HTS are summarized in Table 2. All cell culture procedures were conducted on a clean bench, as previously described [5,24,25]. Both normal and HTS tissues were cut into small pieces of approximately 1–2 mm size, soaked in a dispase II (1 U/mL; Gibco, Waltham, MA, USA) solution, and maintained overnight following a digital rocker at 4 °C. Subsequently, the dermis was separated and digested with collagenase type IV solution (500 U/mL, Gibco) for 60 min at 37 °C. During the digestion, the sample solution was gently shaken every 15 min. Samples were then inactivated using complete medium composed of Dulbecco’s modified Eagle’s medium (DMEM) containing 10% fetal bovine serum (FBS; Biowest, Riverside, MO, USA) and 1% antibiotic–antimycotic with penicillin, streptomycin, and amphotericin B (Gibco). The solutions were filtered and centrifuged at 300× *g* for 5 min. Pellets were resuspended and cultured at 37 °C in 5% CO_2_. The HTSFs at passage two were used for exosome extraction. 

### 4.2. Exosome Purification 

Exosome extraction and isolation were performed as described previously [24,25]. Briefly, NFs and HTSFs at 50–60% confluence were washed twice with DPBS (Biowest) and cultured in complete medium containing 10% exosome-depleted FBS (Gibco) for 48 h. The medium was collected and centrifuged at 300× *g* for 10 min and then again at 16,000× *g* for 30 min to remove any remaining cell debris. The supernatants were transferred to a Macrosep Advance Centrifugal Device with a 100 kDa omega membrane (Pall Corporation, Port Washington, NY, USA) and centrifuged at 4000× *g* for 1 h. The remaining exosomal solution was collected and further centrifuged to minimize volume in the device. Subsequently, exosomes were isolated using an Exo-spinTM Midi Purification Kit (Cell Guidance Systems, Cambridge, UK) according to the manufacturer’s instructions. Samples were mixed with Exo-spin^TM^ buffer in a 1:2 ratio and incubated overnight on a digital rocker at 4 °C. After centrifugation at 16,000× *g* for 1 h, the pellet was dissolved in 1 mL DPBS and loaded on the column. Then, 0.5 mL DPBS was applied to the column and the fraction was collected. This process was repeated a total of 16 times, resulting in 7–16 fractions, which were then concentrated using the aforementioned Centrifugal Device. The total protein concentration of the exosomes was quantified using a bicinchoninic acid (BCA) protein assay kit (Thermo Scientific, Waltham, MA, USA). The exosomes were stored at –80 °C until use. Both NF- and HTSF-exosomes strongly expressed the surface markers CD9, CD63, and CD81, which were detected by Western blotting [24,25]. The size and morphology of the HTSF-exosomes were determined by transmission electron microscopy (TEM) [24] in our previous study. 

### 4.3. Human Melanocyte Culture and Exosome Treatment

NHEM M2 cells were purchased from PromoCell (Heidelberg, Germany) and cultured in melanocyte growth medium, composed of basal medium and supplements (PromoCell), according to the manufacturer’s instructions. The melanocytes were treated with NF-derived exosomes (NF-exo) or HTSF-derived exosomes (SF-exo) at 100 μg/mL [24], and the times were noted, respectively. The control groups were treated with DPBS containing the same volume of DPBS as the exosome-treated groups.

### 4.4. Cell Proliferation Assay

Melanocyte proliferation was assessed using the CellTiter-Glo Luminescent Cell Viability Assay kit (Promega, Madison, WI, USA) as described previously [25]. Melanocytes were seeded at a density of 1.0 × 10^4^ cells in 96-well plates (Corning, New York, NY, USA) and cultured for 48 h. The cells were then treated with exosomes for 10 days at 2-day intervals. After 2-, 6-, and 10-day treatments, 100 µL of CellTiter-Glo reagent was added to the medium and incubated for 10 min at room temperature, respectively. Luminescence was detected using a DTX 880 multimode detector (Beckman Coulter, Fullerton, CA, USA). Viability was calculated as follows: viability (%) = (sample luminescence − background luminescence)/(control sample luminescence − background luminescence) × 100. 

### 4.5. Melanin Content Assay

Melanocytes were seeded at a density of 1 × 10^5^ cells in a 35 mm cell culture dish (Corning) and cultured for 48 h. After 2-day treatment with exosomes, the cell culture medium was collected and centrifuged for 15 min at 1000× *g*, and the supernatants were obtained as test samples. Melanin content in the samples was measured using a human melanin enzyme-linked immunosorbent assay kit (CSB-E14051h, Cusabio Technology, Houston, TX, USA) according to the manufacturer’s instructions. The optical density was measured using an Epoch microplate spectrophotometer at 450 nm (BioTek, Winooski, VT, USA), and the results are presented as the fold change with respect to the levels in DPBS-treated control cells.

### 4.6. Quantitative Reverse Transcription-Polymerase Chain Reaction (qRT-PCR) 

Melanocytes were harvested 2 days after exosome treatment. Total RNA was extracted using the ReliaPrep RNA Miniprep System (Promega) according to the manufacturer’s instructions. RNA concentration was measured using a NanoDrop Spectrophotometer (BioTek, Winooski, VT, USA), and cDNA was synthesized using PrimeScript RT Master Mix (Perfect Real Time) (Takara, Shiga, Japan). Subsequently, 50 ng of cDNA, 0.5 µM primers (Table 3), and a 2 × PCR premix (Takara) were added to a 96-well real-time PCR plate (Roche, Basel, Switzerland) and run in a LightCycler 96 system (Roche) under the following reaction conditions: initial denaturation at 95 °C for 30 s, 40 cycles of amplification at 95 °C for 5 s and 60 °C for 20 s, and extension at 72 °C for 30 s. The mRNA levels of target genes were normalized to the level of GAPDH using the 2^−ΔΔCT^ method (control cells treated with DPBS set as 1.0) [61]. Each qPCR was performed in triplicate using cDNA from three different melanocyte cultures treated with exosomes. 

### 4.7. Western Blot Analysis

Melanocytes were harvested 2 days after exosome treatment and lysed in radioimmunoprecipitation assay buffer containing protease and phosphatase inhibitors (Sigma-Aldrich, St. Louis, MO, USA). The detailed methods have been described previously [25]. Protein concentrations in the lysates were measured using a Pierce BCA Protein Assay Kit (Thermo Fisher Scientific, Carlsbad, CA, USA). Lysates were mixed with 5 × loading sample buffer (GenScript, Piscataway, NJ, USA) and heated for 5 min at 95 °C. Samples were subjected to electrophoresis on 8% or 15% SurePAGE^TM^, Bis–Tris gels (GenScript), electro-transferred onto polyvinylidene difluoride membranes, and blocked with 5% (*w*/*v*) bovine serum albumin (Sigma) or skim milk (BD DIFCO, Franklin Lakes, NJ, USA) in Tris-buffered saline containing 0.1% Tween-20 for 1 h at room temperature. The primary antibodies used are listed in Table 4. The secondary antibodies included horseradish peroxidase (HRP)-conjugated goat anti-rabbit IgG (1:2500; Millipore, Billerica, MA, USA) and HRP-conjugated goat anti-mouse IgG (1:2500; Millipore). To detect the expression of total proteins corresponding to phosphoproteins, the membranes were reused after stripping with stripping solution (ATTO, Tokyo, Japan). Images were obtained using a chemiluminescence imaging system (WSE-6100; ATTO, Tokyo, Japan), and the optical density of the bands was measured using the ImageJ software (Version 1.53, NIH, Bethesda, MD, USA). Protein expression was normalized to that of β-actin; the ratio of Exo-treated cells to DPBS-treated control cells was calculated (cells treated with DPBS as 1.0). Phosphoprotein expression is expressed as a ratio to the corresponding total form.

### 4.8. MicroRNA Analysis

The entire process of MicroRNA (miRNA) analysis was performed by eBiogen Inc. (Seoul, Republic of Korea), as described previously [24]. Briefly, total RNA was extracted from NF-exo and SF-exo. RNA quality was assessed, and library construction and sequencing of control and RNAs samples were performed using the NEBNext Multiplex Small RNA Library Prep kit (New England BioLabs, Ipswich, MA, USA) according to the manufacturer’s instructions. Quantile normalization was used to compare the miRNAs derived from SF-exo and NF-exo. miRWalk 2.0 was used for miRNA target prediction.

### 4.9. Statistical Analyses

SPSS Statistics (version 24.0; SPSS, Inc., Chicago, IL, USA) was used for statistical analyses. Results are presented as means ± standard deviations. Comparisons of the three groups were performed using the Kruskal–Wallis one-way analysis of variance (ANOVA) test. Subsequently, the Mann–Whitney U test was used for pairwise comparisons. Statistical significance was set at *p* < 0.05 or 0.01.

## 5. Conclusions

Our data revealed, for the first time, that HTSF-exosomes suppress the cell growth and melanin synthesis in NHEMs. This effect was attributed to the decreased expression of the transcription factors Pax3, Sox10, and MITF, as well as the melanin synthesis regulatory enzymes TYR, TRP1, and TRP2. Additionally, in NHEMS, several signaling pathways, including the Smad-dependent, Smad-independent, and STAT pathways, were affected by the treatment with HTSF-exosomes. Such molecular evidence may provide new insights into the pathological role of HTSF-exosomes in the hypopigmentation in post-burn HTSs, the development of therapies targeting the exosome production or release from HTSFs, and innovative therapeutic strategies for hypopigmentation in post-burn HTSs. Moreover, HTSF-exosomes represent a promising therapeutic strategy for treating hyperpigmentation disorders. 

## Figures and Tables

**Table 1:** Relative expression of microRNA in HTSF-exosomes.

Gene Symbol	Fold Change
Hsa-let-7a-2-3p	10.2
Hsa-let-7b-3p	20.3
Hsa-let-7e-3p	243.3
Hsa-let-7f-1-3p	33.7
Hsa-let-7i-3p	13.4
Hsa-miR-31-5p	6.0
Hsa-miR-342-3p	8.8
Hsa-miR-125a-5p	11.5
Hsa-miR-365a-5p	73.9
Hsa-miR-125b-5p	5.0
Hsa-miR-138-5p	3.5
Hsa-miR-605-5p	64.9

Results are presented as fold changes with respect to the levels of NF-exosomes. HTSF, hypertrophic scar fibroblast; NF, normal fibroblast.

**Table 2:** Demographic characteristics of patients with post-burn hypertrophic scars.

Patients (n = 4)	Location of Specimens (Scar/Normal)	Age (Years)	Sex	Total Burn Surface Area (%)
1	Arm/arm	38	Male	19
2	Leg/leg	26	Male	11
3	Trunk/trunk	30	Male	20
4	Chest/chest	40	Male	13

**Table 3:** Real-time polymerase chain reaction primer sequences.

Gene	Accession No.	Forward (5′ → 3′)	Reverse (5′ → 3′)
GAPDH	NM_ 002046.7	CATGAGAAGTATGACAACAGC- CT	AGTCCTTCCACGATACCAA- AGTT
BAX	NM_004324.1	CCTTTTGCTTCAGGGTTTCA	CCATGTTACTGTCCAGTTCG
BCL2	NM_000633.3	TGCGGCCTCTGTTTGATTT	AGGCATGTTGACTTCATTGT
CASP3	NM_004346.1	GGAAGCGAATCAATGGACTC- CTGG	GCATCGACATCTGTACCAG- ACC
BIRC2	NM_001166.1	CAGACACATGCAGCTCGAATG- AG	CACCTCAAGCCACCATCAC- AAC
BIRC3	NM_001165.2	GCTTTTGCTGTGATGGTGGACTC	CTTGACGGATGAACTCCTGT- CC
PAX3	NM_181459.1	GGCTTTCAACCATCTCATTCCCG	GTTGAGGTCTGTGAACGGT- GCT
SOX10	NM_006941.1	ATGAACGCCTTCATGGTGTGGG	CGCTTGTCACTTTCGTTCAGCAG
MITF	NM_198159.1	GGCTTGATGGATCCTGCTTTGC	GAAGGTTGGCTGGACAGGA- GTT
TYR	NM_000372.2	GCACAGATGAGTACATGGGAGG	CTGATGGCTGTTGTACTCCT- CC
TYRP1	NM_000550.1	TCTCAATGGCGAGTGGTCTGTG	CCTGTGGTTCAGGAAGACG- TTG
TYRP2	NM_001922.2	CTCAGACCAACTTGGCTACAGC	CAACCAAAGCCACCAGTGT- TCC

**Table 4:** Western blot primary antibody list.

Target	Host	Dilution	Company (Cat. No.)
GAPDH	Rabbit	1:1000	Cell Signaling Technology, Danvers, MA, USA (2118S)
GAPDH	Mouse	1:1000	Santa Cruz Technology, Dallas, TX, USA (sc-47724)
Bax	Rabbit	1:1000	Abcam, Cambridge, UK (ab199677)
Bcl2	Rabbit	1:1000	Abcam (ab196495)
Caspase 3	Rabbit	1:1000	Cell Signaling Technology (9662S)
c-IAP1	Mouse	1:500	Santa Cruz Technology (sc-271419)
c-IAP2	Rabbit	1:1000	Cell Signaling Technology (3130S)
Pax3	Rabbit	1:1000	Cell Signaling Technology (12412S)
Sox10	Mouse	1:500	Santa Cruz Technology (sc-365692)
MITF	Rabbit	1:1000	Cell Signaling Technology (12590S)
Tyrosinase	Mouse	1:500	Santa Cruz Technology (sc-20035)
TRP1	Mouse	1:500	Santa Cruz Technology (sc-166857)
TRP2	Mouse	1:500	Santa Cruz Technology (sc-166717)
Phospho-Smad2	Rabbit	1:1000	Cell Signaling Technology (3108S)
Smad2	Rabbit	1:1000	Abcam (ab33875)
Phospho-Smad3	Rabbit	1:1000	Invitrogen, Waltham, MA, USA (MA5-14936)
Smad3	Rabbit	1:1000	Cell Signaling Technology (9523S)
Phospho-TAK1	Rabbit	1:1000	Cell Signaling Technology (9339S)
TAI1	Rabbit	1:1000	Cell Signaling Technology (5206S)
Phospho-JNK	Rabbit	1:1000	Cell Signaling Technology (9251S)
JNK	Rabbit	1:1000	Cell Signaling Technology (9252S)
Phospho-p38	Mouse	1:1000	Cell Signaling Technology (9216S)
p38	Rabbit	1:1000	Cell Signaling Technology (8690S)
Phospho-ERK	Rabbit	1:1000	Cell Signaling Technology (4370S)
ERK	Mouse	1:1000	Cell Signaling Technology (4696S)
Phospho-STAT1	Rabbit	1:1000	Cusabio Technology, Houston, TX, USA (CSB-PA050162)
STAT1	Rabbit	1:1000	Cusabio Technology (CSB-PA825331)
Phospho-STAT3	Rabbit	1:1000	Abcam (ab76315)
STAT3	Rabbit	1:1000	Cusabio Technology (CSB-PA004173)
Phospho-STAT5	Rabbit	1:1000	Cell Signaling Technology (9351S)
STAT5	Rabbit	1:1000	Cell Signaling Technology (94205S)
Phospho-STAT6	Rabbit	1:1000	Cell Signaling Technology (56554S)
STAT6	Rabbit	1:1000	Cell Signaling Technology (5397S)

## Data Availability

The datasets generated and/or analyzed during the current study are available from the corresponding author upon request.
